# The effect of moving to East Village, the former London 2012 Olympic and Paralympic Games Athletes' Village, on mode of travel (ENABLE London study, a natural experiment)

**DOI:** 10.1186/s12966-020-0916-0

**Published:** 2020-02-10

**Authors:** Elizabeth S. Limb, Duncan S. Procter, Ashley R. Cooper, Angie S. Page, Claire M. Nightingale, Bina Ram, Aparna Shankar, Christelle Clary, Daniel Lewis, Steven Cummins, Anne Ellaway, Billie Giles-Corti, Peter H. Whincup, Alicja R. Rudnicka, Derek G. Cook, Christopher G. Owen

**Affiliations:** 1 Population Health Research Institute, St George’s, University of London, London, UK; 2 Centre for Exercise, Nutrition and Health Sciences, University of Bristol, Bristol, UK; 3 National Institute for Health Research Bristol Biomedical Research Centre, University Hospitals Bristol NHS Foundation Trust and University of Bristol, Bristol, UK; 4 Department of Public Health, Environments and Society, London School of Hygiene and Tropical Medicine, London, UK; 5 MRC/CSO Social and Public Health Sciences Unit, University of Glasgow, Glasgow, UK; 6 NHMRC Centre of Research Excellence in Healthy Liveable Communities, RMIT University, Melbourne, Australia

**Keywords:** Built environment, Travel mode, Physical activity, Walking, Cycling, Housing tenure

## Abstract

**Background:**

Interventions to encourage active modes of travel (walking, cycling) may improve physical activity levels, but longitudinal evidence is limited and major change in the built environment / travel infrastructure may be needed. East Village (the former London 2012 Olympic Games Athletes Village) has been repurposed on active design principles with improved walkability, open space and public transport and restrictions on residential car parking. We examined the effect of moving to East Village on adult travel patterns.

**Methods:**

One thousand two hundred seventy-eight adults (16+ years) seeking to move into social, intermediate, and market-rent East Village accommodation were recruited in 2013–2015, and followed up after 2 years. Individual objective measures of physical activity using accelerometry (ActiGraph GT3X+) and geographic location using GPS travel recorders (QStarz) were time-matched and a validated algorithm assigned four travel modes (walking, cycling, motorised vehicle, train). We examined change in time spent in different travel modes, using multilevel linear regresssion models adjusting for sex, age group, ethnicity, housing group (fixed effects) and household (random effect), comparing those who had moved to East Village at follow-up with those who did not.

**Results:**

Of 877 adults (69%) followed-up, 578 (66%) provided valid accelerometry and GPS data for at least 1 day (≥540 min) at both time points; half had moved to East Village. Despite no overall effects on physical activity levels, sizeable improvements in walkability and access to public transport in East Village resulted in decreased daily vehicle travel (8.3 mins, 95%CI 2.5,14.0), particularly in the intermediate housing group (9.6 mins, 95%CI 2.2,16.9), and increased underground travel (3.9 mins, 95%CI 1.2,6.5), more so in the market-rent group (11.5 mins, 95%CI 4.4,18.6). However, there were no effects on time spent walking or cycling.

**Conclusion:**

Designing walkable neighbourhoods near high quality public transport and restrictions on car usage, may offer a community-wide strategy shift to sustainable transport modes by increasing public transport use, and reducing motor vehicle travel.

## Background

A low level of physical activity poses a serious threat to health due to its association with premature mortality and non-communicable disease [1]. The healthcare cost of low physical activity is high, costing the UK National Health Service (NHS) £0.9 billion in 2006/07 alone [2]. This has led to physical activity recommendations becoming enshrined into health policy, with guidelines generally recommending at least 150 min of moderate-intensity or 75 min of vigorous-intensity activity per week, accompanied by muscle strengthening exercises [1, 3, 4]. In the UK, only a third of men and 40% of women report these recommended levels of activity [5]. However, since recent evidence suggests that even low levels of physical activity can be beneficial for health, particularly being protective against cardiovascular disease, there is a shift towards encouraging everyone, particularly those with low levels of physical activity, to become a little more active [6, 7].

While there is inconclusive evidence that community-wide interventions to increase physical activity are effective [8], walking is a universal form of physical activity available to most people, and strategies to promote walking could yield important health benefits including reducing the risk of obesity [9] and contributing to tackling climate change [10]. Changing the built environment to promote use of public transport may offer a strategy to increase physical activity levels, through increased walking and cycling [11–13], which could potentially impact on health [14–16]. However, the evidence-base is largely cross-sectional, and longitudinal studies are needed to demonstrate cause and effect [17]. Moreover, accuracy in quantifying the active component of everyday travel has been hampered by use of self-reported travel diaries, which can often be imprecise and unreliable [18, 19]. Objective measures are being used increasingly to identify travel modes. By combining data from accelerometers and global positioning system (GPS) monitors, machine learning tools are able to automatically discriminate between different travel modes [20, 21]. For example, we have recently validated such an approach, making use of a gradient boosting machine learning tool [22]. There have been calls for high quality evidence to evaluate the effect of environmental interventions on health behaviours, particularly physical activity, making use of natural experiments where the population effects of change in travel infrastructure can be examined [23–25]. However, sizeable alterations in travel infrastructure and studies with sufficient numbers are needed to demonstrate change in active travel behaviour, and given the practical difficulties and high costs involved in making marked changes to the built environment, few opportunities arise [26]. In addition, the degree of change should ideally be compared with a control population who are similar socio-demographically but not exposed to the same change in travel infrastructure [27], and analysis conducted within a rapid time scale to distinguish any potential effects (favourable or otherwise) from underlying trends in physical activity levels over time.

The East Village neighbourhood, the former London 2012 Olympics Athletes’ Village offered an opportunity for just such a natural experiment [28]. East Village is a purpose-built mixed-use residential development, and was built on active design principles specifically to encourage active living, by improving neighbourhood walkability and access to public transport and open space with restrictions on vehicle parking [28] Utilising Geographic Information Systems (GIS) allows objective and detailed characterisation of built environment features at baseline and follow-up, including the ability to quantify changes in walkability, access to green and public open space, and access to public transport facilities. Adults seeking to move into differently tenured accommodation in East Village (social, intermediate / affordable, and market-rent), were recruited and followed-up after 2 years, once half had relocated to East Village [28]. The present study examines changes in travel mode (walking, cycling, motorised vehicle and train use) using objective measures in those who moved to East Village compared with those who did not.

## Methods

Between January 2013 and December 2015, the Examining Neighbourhood Activities in Built Living Environments in London (ENABLE London) study recruited adults seeking to move into three different housing tenures in East Village: social housing, recruited by East Thames Group Housing Association; intermediate housing (affordable market-rent / shared ownership / shared equity), recruited by Triathlon Homes; and market-rent, recruited by Get Living London [28]. For social housing, eligible participants were those currently living in or on a waiting list for social housing in the London Borough of Newham. Priority was based on a points system which included current living conditions, earnings and health circumstances. Unfortunately, the points scoring system could not be shared with the investigators. Eligibility for intermediate accommodation was based on a rigorous financial process – they were required to be first-time buyers, living or working in London and with an annual household income less than £66,000 for 1- or 2-bedroom homes or less than £80,000 for 3-bedroom homes. There were no financial restrictions for those seeking market-rent accommodation. Those applying to move into East Village social housing were provided with information about the study and invited to take part by East Thames Group representatives directly, while the ENABLE team (in association with Triathlon Homes and Get Living London) invited those from intermediate and market-rent groups. Those who agreed to take part and subsequently chose to move to East Village were exposed to the intervention. Those who took part but did not move to East Village, choosing to stay in their current housing or move elsewhere, represented the Control group. Follow-up of the cohort was carried out after 2 years from January 2015 to December 2017 after half had moved to East Village. Assessments at both time points were similar and carried out during home visits by a team of trained fieldworkers (with 1 or more participants per household); details of the assessments have been described elsewhere [28]. In brief, these included individual questionnaires and objective measures of physical activity levels with concurrent recording of geographic location over a 7-day period. Details of these measures are provided below.

### Physical activity and geographic location

At baseline and follow-up, participants were asked to wear an ActiGraph GT3X+ accelerometer (ActiGraph LLC, Florida, USA) and a GPS receiver (Qstarz BT-1000XT; Taipei, Taiwan), set to record location every 10 s, on an elasticated belt around the waist during waking hours for 7 consecutive days. Accelerometers provided objective measures of daily physical activity, which have been previously validated against gold standard assessment of energy expenditure [29], and more recently, against oxygen consumption [30]. Combined ActiGraph accelerometer and GPS travel recorder data were analysed using a previously described automated machine learning algorithm, which allocated each 10-s epoch of combined data to one of four travel modes, quantifying the daily time spent (i) walking, (ii) cycling, (iii) traveling by motorised vehicle (including car/van/bus/motorbike) or (iv) overground train. A fifth category classified recorded time where a journey was not taking place and the participant was stationary, e.g., sitting indoors at home or at work or stationary outside [22]. Gaps in the data due to loss of GPS signal were further classified as “underground” if the GPS signal was lost or regained within close proximity (200 m) of an underground station, and the time lapse between loss and regained signal was from 2 min to 2 h. However, as underground trains in the London transport system also run above ground, so there was potential for misclassification between “underground” and “overground train” modes of travel. The 10-s epoch data were then summed to provide daily minutes in each travel mode and total daily GPS minutes. Walking and cycling minutes were also combined to provide a measure of “active travel”. To minimise bias due to low wear time of the GPS monitor or low GPS recording activity, days were only included in the analysis if there was a corresponding day of ≥540 min of valid accelerometry data, in line with the criteria specified a priori for the main accelerometry outcomes.

### Environmental exposures

Participants were geocoded to the centroid of the footprint of their building of residence at both baseline and follow-up. At both time points, participants were assigned the value of the closest available Public Transport Accessibility Level (PTAL) score [31] from their home address, as a measure of accessibility to public transport. Measures of neighbourhood walkability provided a relative index, derived by combining scores from three different domains; (i) land-use mix (as a measure of residential, commercial, office, entertainment and institutional building footprints), (ii) street connectivity (from the number of 3 or more branch road junctions), and (iii) residential density, within a within a 1 km street network home address-centred buffer using Ordnance Survey (OS) data [32]. A park proximity variable was computed as the shortest street network distance from the residential addresses to the nearest entrance of the closest park. Using data from Greenspace Information for Greater London (GiGL) [33], three types of park were considered (Metropolitan, District and Local), based on their size and the number and type of facilities available, as described in the Greater London Authority (GLA) reference plan [34].

### Covariates

Laptop-based self-completion questionnaires were used to collect data on age, sex, self-defined ethnicity, work status, occupation and car/van ownership. Participants were categorised as ‘White’, ‘Asian’, ‘Black’, ‘Mixed’, or ‘Other’; the latter two categories were combined in the analysis. Occupation based socioeconomic status was coded using the National Statistics Social-Economic Classification (NS-SEC) to code participants into ‘higher managerial or professional occupations’, ‘intermediate occupations’, and ‘routine or manual occupations’. [35] An additional ‘economically inactive’ category included those seeking employment, unable to work due to disability or illness, retired, looking after home and family, and students [36]. Two neighbourhood perception scores, measuring crime (i.e., vandalism, feeling unsafe to walk in neighbourhood, presence of threatening groups) and neighbourhood quality (i.e., accessible features, attractiveness, and enjoyment of living in neighbourhood), were derived at baseline using exploratory factor analysis on 14 neighbourhood perception items in the questionnaire [37, 38], and the same items were used to obtain scores at follow-up.

### Statistical analysis

For each motion category (travel mode, underground and stationary), average daily minutes at baseline were derived using multi-level linear regression models (level 1 was day within individual and level 2 was individual). Daily was regressed on day-order-of-wear, day-of-week and month-of-wear as fixed effects and participant as a random effect to allow for up to 7 days recording for each individual. The mean of the within-person residuals for each participant was obtained and added to the population mean to produce an unbiased average daily estimate for each participant. This was repeated for the follow-up data. The change in average daily minutes from baseline to follow-up for each travel mode was then examined using multilevel linear regression models, where level 1 was individual and level 2 was household. For each travel mode, average daily minutes at follow-up was regressed on average daily minutes at baseline adjusting for East Village/Control group, sex, age group, ethnic group and housing group as fixed effects, and household as a random effect. The regression coefficient for East Village/Control group thus provided an estimate of the average within-person change in the East Village group compared with the average within-person change in the Control group, minimising bias and maintaining power. Stratified models by housing group examined the effects in the different housing groups. Checks were carried out to confirm that the distribution of residuals from the models were normally distributed. Sensitivity analyses were carried out for the GPS outcomes: (i) restricting analyses to those who were working or studying at baseline; (ii) repeating analyses for weekdays only and weekend days only; (iii) multiple imputation methods to assess the impact of missing data from those who provided GPS data at baseline but not at follow-up. STATA *mi impute* commands were used with linear regression models and 40 imputations to impute GPS outcome data, conditional on the model variables (baseline GPS outcome, East Village / Control group, sex, age group, ethnic group and housing group).

## Results

At baseline, 1063/1278 adults (83%) provided GPS data of whom 991 also provided at least one corresponding day of ≥540 min accelerometer wear time. At follow-up, 877 adults were re-examined, half had moved to East Village; 714 (81%) provided GPS data, 681 with at least one corresponding day with ≥540 min accelerometry wear time. Longitudinal analyses were restricted to 578 who had valid GPS data at both baseline and follow-up and baseline characteristics for these 578 adults are shown in Table 1 by East Village/Control group and housing group. Age and sex patterns were similar to those for both the 877 who were followed-up and the 1278 recruited at baseline [39]. However, those with complete GPS data were more likely to be of white ethnic origin and higher managerial, professional or intermediate occupations. In the social housing group, the East Village and Control group were similar in age, sex and socio-economic distributions, but the East Village group were more likely to be of black ethnic origin. In the intermediate group, the East Village group were more likely to be younger, male, of white ethnic origin and economically active. In the market-rent group, age, sex, ethnic group and socio-economic status were similar in the East Village and Control groups. There was no difference between the East Village group and Control group in the proportion of households who owned a car at baseline. Slightly fewer of the East Village group were working at baseline and 24% were classified as economically inactive compared with 18% of the Control group. Using public transport to travel to work or study was reported more frequently amongst those who subsequently moved into East Village (*p* = 0.004 for all housing groups combined). Use of private transport and walking/cycling for travel to work or study were similar in the East Village and Control groups, although social housing participants were more likely to use private transport and less likely to walk/cycle compared with intermediate and market-rent participants. Time spent in the different GPS motion categories were similar in the East Village and Control groups, although the Control group recorded slightly higher mean walking and cycling minutes at baseline. The intermediate and market-rent housing groups recorded more walking, cycling, overground and underground train minutes and fewer vehicle minutes compared with the social housing group.

**Table 1:** Baseline characteristics and GPS outcomes for those with GPS data at baseline and follow-up

N	All housing groups		*p*-value	Social housing group		Intermediate housing group		Market rent housing group	
	Control 285	East Village 293		Control 74	East Village 127	Control 141	East Village 142	Control 70	East Village 24
	n (%)	n (%)		n (%)	n (%)	n (%)	n (%)	n (%)	n (%)
Age			0.005						
16–24	44 (15%)	68 (23%)		7 (9%)	26 (20%)	20 (14%)	29 (20%)	17 (24%)	13 (54%)
25–34	116 (41%)	133 (45%)		18 (24%)	34 (27%)	65 (46%)	93 (65%)	33 (47%)	6 (25%)
35–49	100 (35%)	80 (27%)		44 (59%)	57 (45%)	46 (33%)	19 (13%)	10 (14%)	4 (17%)
50+	25 (9%)	12 (4%)		5 (7%)	10 (8%)	10 (7%)	1 (1%)	10 (14%)	1 (4%)
Sex: female	173 (61%)	159 (54%)	0.12	58 (78%)	91 (72%)	82 (58%)	57 (40%)	33 (47%)	11 (46%)
Ethnic group			0.001						
White	152 (53%)	161 (55%)		17 (23%)	24 (19%)	87 (62%)	117 (82%)	48 (69%)	20 (83%)
Black	48 (17%)	78 (27%)		22 (30%)	75 (59%)	21 (15%)	3 (2%)	5 (7%)	0 (0%)
Asian	57 (20%)	30 (10%)		29 (39%)	13 (10%)	23 (16%)	15 (11%)	5 (7%)	2 (8%)
Other	28 (10%)	24 (8%)		6 (8%)	15 (12%)	10 (7%)	7 (5%)	12 (17%)	2 (8%)
NS-SEC ^a^			0.03						
Higher managerial/professional	163 (57%)	130 (45%)		17 (23%)	16 (13%)	97 (69%)	99 (70%)	163 (70%)	15 (63%)
Intermediate occupations	43 (15%)	56 (19%)		12 (16%)	21 (17%)	18 (13%)	29 (21%)	13 (19%)	6 (25%)
Routine/manual occupations	29 (10%)	33 (11%)		16 (22%)	24 (19%)	11 (8%)	9 (6%)	2 (3%)	0 (0%)
Economically inactive	50 (18%)	71 (24%)		29 (39%)	64 (51%)	15 (11%)	4 (3%)	6 (9%)	3 (13%)
Car or van in household	111 (46%)	104 (46%)	0.96	31 (53%)	47 (51%)	55 (46%)	49 (43%)	25 (41%)	8 (40%)
Currently working or studying	245 (86%)	240 (82%)	0.18	51 (69%)	80 (63%)	128 (91%)	138 (97%)	66 (94%)	22 (92%)
Mode of travel to or from work / study (not mutually exclusive)									
Public transport	156 (67%)	182 (79%)	0.004	31 (66%)	56 (74%)	83 (67%)	110 (82%)	42 (67%)	16 (76%)
Private car/motorbike/taxi	35 (15%)	28 (12%)	0.36	10 (21%)	16 (21%)	19 (15%)	10 (7%)	6 (10%)	2 (10%)
Walk/cycle	141 (61%)	128 (55%)	0.27	19 (40%)	40 (53%)	82 (67%)	77 (57%)	40 (63%)	11 (52%)
GPS motion category (minutes)	mean (sd)	mean (sd)	*p*-value	mean (sd)	mean (sd)	mean (sd)	mean (sd)	mean (sd)	mean (sd)
Walking	39.7 (25.9)	37.7 (24.5)	0.35	30.6 (21.4)	31.3 (25.0)	40.9 (24.3)	43.3 (22.2)	46.7 (30.5)	38.2 (27.6)
Cycling	6.1 (13.2)	3.5 (6.7)	0.003	2.3 (2.7)	2.5 (4.1)	7.0 (13.6)	4.7 (8.6)	8.2 (17.5)	2.1 (3.6)
Walking + cycling	45.8 (30.0)	41.3 (26.4)	0.06	32.9 (22.0)	33.8 (25.8)	48.0 (28.4)	48.1 (24.9)	54.9 (35.8)	40.2 (28.1)
Motorised vehicle	37.3 (37.7)	38.0 (38.9)	0.84	42.6 (47.0)	48.8 (47.5)	36.5 (31.5)	29.4 (27.9)	33.5 (38.0)	31.6 (30.5)
Overground train	14.6 (21.3)	14.5 (18.9)	0.98	8.3 (12.2)	8.5 (13.7)	16.5 (22.6)	20.4 (22.0)	17.4 (24.8)	11.6 (11.7)
Underground train ^b^	14.3 (17.5)	15.1 (17.5)	0.55	11.6 (20.2)	8.0 (14.8)	14.9 (16.7)	20.9 (18.0)	15.8 (15.6)	18.9 (15.0)
Stationary, incl inside & outside	440 (193)	449 (188)	0.58	507 (192)	496 (184)	421 (190)	418 (179)	407 (184)	380 (207)
Total GPS time	552 (207)	558 (195)	0.73	602 (201)	596 (190)	537 (206)	537 (188)	529 (210)	482 (225)

^a^Economically inactive includes those not part of the labour force who are unemployed or not available for work, including students and house carers

^b^Underground minutes are assumed from portions of missing GPS signal where the GPS signal is lost within 200 m of an underground station and regained within 200 m of a different underground station

Table 2 shows the change in neighbourhood perception and built environment variables for the East Village and Control groups. Compared with baseline data, those participants who had moved to East Village showed significant improvements in their built environments, living closer to their nearest park (on average living 547 m closer), improved access to public transport and living in a more walkable area (with appreciable increases in walkability). They also reported more positive perceptions of their local area, with improved perceived neighbourhood crime and quality scores. These differences were most marked for social housing participants.

**Table 2:** Change in measures of the objective built environment and neighbourhood perceptions from baseline to follow-up

	All housing groups			Social			Intermediate			Market-rent		
	mean	(95% CI)	*p*-value	mean	(95% CI)	*p*-value	mean	(95% CI)	*p*-value	mean	(95% CI)	*p*-value
Objective built environment characteristics												
Control group	*N* = 256			*N* = 73			*N* = 127			*N* = 56		
Distance to closest park (m) ^a^	28	(−23, 78)	0.28	27	(−44, 97)	0.45	31	(−39, 101)	0.39	21	(− 123, 166)	0.77
Access to public transport (PTAL) ^b^	−0.1	(−0.3, 0.1)	0.35	− 0.2	(− 0.5, 0.1)	0.20	− 0.1	(− 0.4, 0.3)	0.63	0.0	(− 0.5, 0.4)	0.94
Walkability ^c^	0.3	(0.0, 0.6)	0.09	−0.2	(− 0.7, 0.3)	0.38	0.5	(0.0, 1.0)	0.04	0.3	(−0.2, 0.9)	0.25
Land use mix ^d^	0.02	(0.00, 0.04)	0.08	−0.02	(− 0.05, 0.01)	0.22	0.04	(0.01, 0.08)	0.03	0.02	(−0.02, 0.06)	0.25
Residential density ^e^	1.8	(1.0, 2.7)	< 0.001	1.2	(0.2, 2.3)	0.016	2.1	(0.7, 3.5)	0.003	2.0	(0.2, 3.8)	0.03
Street connectivity ^f^	−0.1	(−0.2, 0.1)	0.38	−0.2	(−0.4, 0.0)	0.13	0.0	(−0.2, 0.2)	0.93	−0.1	(−0.4, 0.2)	0.63
East Village group	*N* = 277			*N* = 124			*N* = 130			*N* = 23		
Distance to closest park (m) ^a^	−547	(− 597, − 498)	< 0.001	− 463	(− 529, − 397)	< 0.001	− 602	(− 673, − 530)	< 0.001	− 693	(− 952, − 435)	< 0.001
Access to public transport (PTAL) ^b^	1.2	(0.9, 1.5)	< 0.001	2.0	(1.5, 2.5)	< 0.001	0.7	(0.2, 1.1)	0.007	0.3	(−0.9, 1.5)	0.61
Walkability ^c^	2.4	(2.1, 2.7)	< 0.001	2.7	(2.3, 3.1)	< 0.001	2.0	(1.5, 2.5)	< 0.001	2.4	(0.4, 4.3)	0.02
Land use mix ^d^	0.38	(0.36, 0.41)	< 0.001	0.40	(0.37, 0.43)	< 0.001	0.37	(0.34, 0.41)	< 0.001	0.35	(0.23, 0.47)	< 0.001
Residential density ^e^	14.4	(12.8, 16.0)	< 0.001	14.7	(12.3, 17.0)	< 0.001	13.3	(11.0, 15.6)	< 0.001	19.1	(12.4, 25.9)	< 0.001
Street connectivity ^f^	−1.1	(−1.2, −0.9)	< 0.001	−0.9	(−1.1, −0.7)	< 0.001	−1.2	(−1.4, −1.0)	< 0.001	−1.2	(−2.0, − 0.4)	0.006
Neighbourhood perceptions ^g^												
Control group	*N* = 285			*N* = 74			*N* = 141			N = 70		
Crime score	0.6	(0.1, 1.0)	0.02	1.3	(0.4, 2.2)	0.005	0.0	(−0.7, 0.7)	1.00	1.0	(0.0, 1.9)	0.04
Quality score	0.7	(0.2, 1.2)	0.01	1.5	(0.6, 2.4)	0.001	0.1	(−0.6, 0.9)	0.73	0.9	(0.0, 1.9)	0.06
East Village group	*N* = 293			N = 127			*N* = 142			*N* = 24		
Crime score	4.7	(4.1, 5.2)	< 0.001	6.1	(5.2, 7.1)	< 0.001	3.5	(2.8, 4.2)	< 0.001	3.8	(1.7, 5.9)	0.001
Quality score	7.0	(6.4, 7.5)	< 0.001	7.9	(7.0, 8.7)	< 0.001	6.3	(5.5, 7.1)	< 0.001	6.5	(5.1, 7.9)	< 0.001

^a^Distance to closest park from choice of local, district and metropolitan parks

^b^PTAL is a Transport for London (TfL) score assessing the availability of public transport options. A positive change indicates improved public transport links

^c^Walkability: The sum of three z-transformed variables, land use mix, residential density and street connectivity. A positive change indicates improved walkability

^d^Land use mix: The heterogeneity with which five functionally different land uses (residential, commercial, office, entertainment and institutional) are co-located in space. Values are normalised between 0 and 1 where 0 indicates single use and 1 indicates a perfectly even distribution of square footage across the different types of land use. A positive change indicates improved land use mix

^e^Residential density: The number of residential units (RU) per km^2^ of land devoted to residential use, including residential building footprint and attached gardens, expressed in 1000 RU/km^2^

^f^Street connectivity: The number of intersections per km of road

^g^Neighbourhood perception scores from exploratory factor analysis on 14 neighbourhood perception items in the questionnaire. A positive score indicates perception of less crime and higher quality in the neighbourhood

The effect of moving to East Village on time spent in different travel modes is shown in Table 3, and summary data in Additional file 1: Table S1. Overall, there was little change in participants’ walking or cycling minutes. However, vehicle minutes decreased on average by 8.3 min per day, with greater effects in the intermediate housing group (9.6 min decrease, 95% CI − 16.9 to − 2.2, *p* = 0.01), and time spent traveling by underground increased by 3.9 min, particularly in the market-rent housing group (11.5 min, 95% CI 4.4 to 18.6, *p* = 0.001). There were large decreases in the East Village group in both stationary and total minutes of recorded time. These varied by housing group, the largest decreases recorded by the social housing group and the smallest decrease by the market-rent housing group. Manual inspection of the data suggested that this was due to reductions in indoor recording among the East Village group, with the GPS signal being blocked by East Village housing. Restricting the analysis to those who were working or studying at baseline gave broadly similar results (Additional file 2: Table S2), although the differences were more marked in the social housing group where one third of the group were not working or studying at baseline. Analysis of weekdays and weekend days (Additional file 3: Table S3) showed similar patterns to the “all days” analyses, although the decrease in vehicle minutes was greater at the weekend. Imputation analyses for those with GPS data at baseline but who did not provide GPS data at follow-up (*n* = 131) gave similar effect size estimates to the complete case analyses (data not shown).

**Table 3:** Change in daily minutes of each GPS motion category in East Village group relative to change in Control group, overall and by housing group

GPS motion category	All housing groups^(a,b)^			Social^(a)^			Intermediate^(a)^			Market rent^(a)^		
	*N* = 578			*N* = 201			*N* = 283			*N* = 94		
	Difference	(95% CI)	*p*-value	Difference	(95% CI)	*p*-value	Difference	(95% CI)	*p*-value	Difference	(95% CI)	*p*-value
Walking	−1.4	(− 5.3, 2.5)	0.48	− 1.8	(− 8.4, 4.8)	0.59	− 1.9	(− 7.5, 3.8)	0.52	− 4.2	(− 15.4, 7.0)	0.46
Cycling	1.1	(−0.5, 2.7)	0.17	0.3	(−0.8, 1.4)	0.58	1.0	(−1.6, 3.7)	0.44	1.9	(−3.6, 7.4)	0.50
Walking + cycling	−0.4	(−4.7, 3.8)	0.85	−1.5	(−8.4, 5.4)	0.68	−0.9	(−7.1, 5.4)	0.78	−2.4	(−16.0, 11.3)	0.74
Motorised vehicle	−8.3	(−14.0, − 2.5)	0.01	−6.2	(− 18.9, 6.6)	0.34	−9.6	(− 16.9, − 2.2)	0.01	− 6.9	(− 19.9, 6.1)	0.30
Overground train	−0.8	(−3.7, 2.2)	0.62	− 1.7	(−5.8, 2.4)	0.41	− 1.5	(− 6.0, 3.0)	0.51	0.2	(−10.1, 10.6)	0.97
Underground train^(c)^	3.9	(1.2, 6.5)	0.005	4.0	(−0.1, 8.1)	0.06	1.6	(−2.7, 6.0)	0.46	11.5	(4.4, 18.6)	0.001
Stationary	− 247	(− 279, − 214)	< 0.001	−354	(− 405, − 304)	< 0.001	− 205	(− 252, − 157)	< 0.001	−127	(− 217, − 38)	0.01
Total GPS minutes	− 253	(− 288, − 217)	< 0.001	− 357	(− 412, − 302)	< 0.001	−217	(− 269, − 164)	< 0.001	− 126	(− 223, − 29)	0.01

^a^All models are adjusted for sex, age group, ethnic group as fixed effects and household as a random effect in a multi-level model

^b^The model for all housing group additionally adjusts for housing tenure group as a fixed effect

^c^Underground minutes are assumed from portions of missing GPS signal where the GPS signal is lost within 200 m of an underground station and regained within 200 m of a different underground station

## Discussion

Using a novel automated approach of identifying mode of travel from combined accelerometry and GPS data, we found at two-year follow-up there was no change in the time spent walking or cycling among those who moved to East Village compared with those living elsewhere. However, the results suggested that vehicle travel had decreased, particularly in the intermediate housing group, and that underground train travel had increased, more so in the market-rent group. While use of underground train also appeared to increase among the social housing group, there appears to be little change in time spent walking.

Our finding of increased public transport underground train use and decreased vehicle use associated with the East Village development fits with a small number of other longitudinal studies carried out in the UK, which have used natural experiment study designs to examine change in travel mode and physical activity levels associated with improved travel infrastructure. Such studies have shown that town-level infrastructure initiatives to encourage active travel, in particular cycling, resulted in modest increases in self-reported cycling prevalence (with an increase from 6 to 7% over a decade) and decreased car travel [40]. Also that living closer to transport infrastructure that created new and enhanced existing walking and cycling routes across the UK, increased self-reported active travel and total physical activity after 2 years compared with those living further away [41]. However, in this study, it is noteworthy that there was no evidence of effect after 1 year and that living closer to the infrastructure was the main determinant of use at 2 years [41], more so than any other theorised cognitive mechanisms of effect [42]. Another UK example of a natural experiment which has shown that change in travel infrastructure can alter travel behaviour, includes the installation of the Cambridge Guided Busway with a traffic free cycleway and walkway, which resulted in greater self-reported weekly cycle and active commuting times [17]. Conversely, changes in parking policy to encourage car use in the work place (with free parking and fewer restrictions) resulted in increased motor vehicle trips and reduced walking and cycling [43]. This is in contrast to our study where car parking restrictions among those living in East Village led to a decrease in car travel, when compared with a control group without such restrictions. However, whilst our findings are commensurate with these other findings, differences in study methods, particularly in characterising modes of travel, do not allow for direct comparisons. Self-reported methods usually report prevalence and the type of trips taken, whereas our objective measures allow the amount of time spent in different travel modes to be quantified. Moreover, although we showed increased use of underground trains and decreased vehicle use associated with moving to East Village, there was no evidence of an effect on levels of walking, cycling or overall measures of physical activity [39]. This raises the possibility of compensatory effects where close proximity to public transport encourages use, but in turn decreases the amount of activity needed to access it. Similar compensatory effects could occur among those moving to East Village, where better walkability/closer proximity to facilities (such as parks, shops, etc.) encourage active mode of travel, but reduce the time spent travelling to reach them. Further analyses of qualitative data from this study could be used to explore these possibilities. Moreover, there is more research required on thresholds of proximity that maximise the health benefits that can be achieved through city planning and urban design.

The ENABLE London study has a number of strengths and limitations worthy of further consideration. A major strength was the clear evidence of positive change in objective measures of the built environment and travel infrastructure associated with moving to East Village, particularly in comparison with the control group who did not move or moved elsewhere and showed little or no change. In particular, the sizeable improvements in access to public transport associated with moving to East Village highlights the legacy of the Olympic Delivery Authority’s transport plan for the London 2012 Olympic and Paralympic Games [44], in addition to marked increases in walkability and closer proximity to a local park (by ½ km or more), as well as sizeable improvements in neighbourhood perceptions of safety and quality. The robust longitudinal study design, targeted those who were seeking to move, minimising potential biases that may have occurred by including those who were not seeking to move who may have had potentially different health behaviours [39]. A unique strength of the ENABLE London study is the social diversity of participants, with representation from three housing groups (social, intermediate and market-rent housing), which allowed social gradients in effects on travel mode associated with moving to East Village to be gauged. To date there has been a dearth of studies that have directly examined or reported on social disparities in interventions to promote active travel, particularly those that have examined the effects of change in travel infrastructure [45, 46]. While power to examine effects across social sub-groups was limited, this study allays concerns over whether such interventions widen social inequalities, in that effects of moving to East Village were broadly similar and in the same direction across the housing groups. This was despite marked differences in travel mode and physical activity levels between housing groups at baseline [38], showing potential benefits for all. Another major strength was the use of an automated machine learning approach, combining accelerometry and GPS data, to measure travel mode allowing more data points to contribute to the analyses, increasing statistical power to establish the presence or absence of effects. The algorithm has been described previously and has major advantages over previously used manual approaches, which are prohibitively labour intensive particularly in larger studies [22].

### Limitations

Misclassification and overlap between overground and underground train travel is possible, which may lead to underestimation of effects but is likely to affect East Village and controls equally. It was also not possible to reliably distinguish public transport bus travel from car travel, thus making it difficult to accurately quantify use of all forms of public transport. Another limitation was the reduced sample size of combined ActiGraph and GPS wear time, due to participants not providing GPS data or not having equivalent days of ≥540 min accelerometer wear time. Participants were asked to re-wear their accelerometers if they didn’t provide 4 days adequate data, but were not asked to re-wear their GPS monitors to encourage compliance for accelerometry as the main outcome for the ENABLE London study. The GPS monitors required charging overnight, and it’s possible that some participants didn’t wear or activate their GPS monitors each day. GPS monitors rely on being able to transmit a signal and it is known that this can be reduced indoors, particularly in blocks of flats rather than individual houses. The loss of GPS signal was particularly noticeable at follow-up among those living in the East Village homes, a high-rise urban environment, affecting both total GPS minutes and GPS minutes classified as stationary. Manual inspection of the GPS data indicated that the GPS signal was lost within close proximity of East Village accommodation blocks, and reappeared at a similar location, suggesting entering and exiting the accommodation block. The decreased stationary time associated with a blocked signal was therefore most likely to be indoor stationary time whilst the participants were in their homes. For example, in the social housing group, the reduction in stationary and total GPS minutes was much greater in those not working or studying at baseline (*n* = 70/201) i.e., those participants who are more likely to be spending longer periods of time at home. However, this loss of GPS signal inside the East Village homes will not have affected the quantification of outdoor modes of travel / motion categories (i.e., outdoor stationary time). It was not possible to test this prior to the study as East Village was not built, but future studies may wish to check GPS signal in potential indoor intervention areas to avoid such difficulties. It should be noted that loss of signal associated with underground travel did not affect the recording of activities, as proximity (within 200 m) to known locations of stations allowed for these activities to be included, despite the loss of signal.

While there was no clear evidence of an effect of moving to East Village on overall physical activity levels, there are other potential health and environmental implications of increased public transport use and decreased car use worthy of consideration, particularly where small shifts in travel mode across a whole population are observed. The extent to which this was due to urban design, or policies to restrict motor vehicle ownership is unclear. Moreover, it is plausible that walkable neighbourhoods with very close proximity to public transport and amenities, decreases active transport, and more research is required to understand how to optimise urban design standards for proximity. Strategies to increase active travel could impact on air quality leading to more environmentally sustainable communities [47, 48]. However, these gains need to be offset against increased use of public transport, particularly use of London underground, where individual exposure to air pollution is high (particularly to small particulate matter, PM_2.5_) with potentially adverse health consequences [49]. This is of particular relevance to this population who are much more likely to use public transport (given London’s extensive transport network system) compared with National Travel Survey data where only 8% use public transport nationally (of which half use the bus) [11]. Increased individual exposure to underground air pollution needs to be weighed against greater environmental sustainability at a population level, in order to fully appreciate the ramifications of future travel infrastructure and policy initiatives. However, this study provides an important addition to the literature providing longitudinal evidence that major investment in travel infrastructure, combined with motor vehicle parking policies, can offer a community-wide strategy to shift transport behaviours towards more sustainable ones.

## Supplementary information

**Additional file 1: Table S1.** Summary data for minutes spent in different GPS motion categories, by East Village/Control group and housing group.

**Additional file 2: Table S2.** Change in daily minutes of activity measured by GPS in East Village group relative to change in Control group, for those who were working or studying at baseline.

**Additional file 3: Table S3.** Change in daily minutes of activity measured by GPS in East Village group relative to change in Control group, for weekdays and weekend days.

## Data Availability

Further details of the ENABLE London study are available from the study website (http://www.enable.sgul.ac.uk/). We welcome proposals for collaborative projects. For general data sharing inquiries, contact Professor Owen (cowen@sgul.ac.uk).

## Abbreviations

ENABLE London: Examining Neighbourhood Activities in Built Living Environments in London
GIS: Geographic Information System
GPS: Global Positioning System
NHS: National Health Service (UK)
NS-SEC: National Statistics Social-Economic Classification (UK)
OS: Ordnance Survey (UK)
PTAL: Public Transport Accessiblity Level score (Transport for London, UK)
