# Supplementary material for: The effect of moving to East Village, the former London 2012 Olympic and Paralympic Games Athletes' Village, on mode of travel (ENABLE London study, a natural experiment)
Source: Int J Behav Nutr Phys Act. 2020 Feb 10;17:15. doi: 10.1186/s12966-020-0916-0 (PMC7011441; doi:10.1186/s12966-020-0916-0)
Supplement: Supplementary file 1 — Additional file 1: Table S1. Summary data for minutes spent in different GPS motion categories, by East Village/Control group and housing group. [file 12966_2020_916_MOESM1_ESM.docx]

**S1 Table. Summary data for minutes spent in different GPS motion categories, by East Village/Control and housing group.**

|  |  | **All housing groups** | | | | **Social** | | | | **Intermediate** | | | | **Market-rent** | | | |
| --- | --- | --- | --- | --- | --- | --- | --- | --- | --- | --- | --- | --- | --- | --- | --- | --- | --- |
|  |  | **Control** | | **East Village** | | **Control** | | **East Village** | | **Control** | | **East Village** | | **Control** | | **East Village** | |
| **N** |  | 285 | | 293 | | 74 | | 127 | | 141 | | 142 | | 70 | | 24 | |
|  |  |  |  |  |  |  |  |  |  |  |  |  |  |  |  |  |  |
| **GPS motion category** | | **Mean** | **(sd)** | **Mean** | **(sd)** | **Mean** | **(sd)** | **Mean** | **(sd)** | **Mean** | **(sd)** | **Mean** | **(sd)** | **Mean** | **(sd)** | **Mean** | **(sd)** |
|  |  |  |  |  |  |  |  |  |  |  |  |  |  |  |  |  |  |
| Walking | Baseline | 40 | (26) | 38 | (24) | 31 | (21) | 31 | (25) | 41 | (24) | 43 | (22) | 47 | (31) | 38 | (28) |
|  | Follow-up | 39 | (25) | 37 | (23) | 33 | (24) | 33 | (21) | 40 | (25) | 41 | (24) | 46 | (24) | 39 | (18) |
|  |  |  |  |  |  |  |  |  |  |  |  |  |  |  |  |  |  |
| Cycling | Baseline | 6 | (13) | 4 | (7) | 2 | (3) | 2 | (4) | 7 | (14) | 5 | (9) | 8 | (18) | 2 | (4) |
|  | Follow-up | 6 | (12) | 5 | (10) | 3 | (2) | 3 | (4) | 6 | (11) | 6 | (13) | 9 | (16) | 7 | (11) |
|  |  |  |  |  |  |  |  |  |  |  |  |  |  |  |  |  |  |
| Walking + cycling | Baseline | 46 | (30) | 41 | (26) | 33 | (22) | 34 | (26) | 48 | (28) | 48 | (25) | 55 | (36) | 40 | (28) |
|  | Follow-up | 45 | (29) | 43 | (26) | 36 | (25) | 36 | (23) | 46 | (29) | 48 | (28) | 54 | (31) | 46 | (24) |
|  |  |  |  |  |  |  |  |  |  |  |  |  |  |  |  |  |  |
| Motorised vehicle | Baseline | 37 | (38) | 38 | (39) | 43 | (47) | 49 | (48) | 36 | (32) | 29 | (28) | 34 | (38) | 32 | (30) |
|  | Follow-up | 38 | (36) | 31 | (40) | 45 | (41) | 41 | (51) | 37 | (36) | 22 | (27) | 33 | (29) | 28 | (26) |
|  |  |  |  |  |  |  |  |  |  |  |  |  |  |  |  |  |  |
| Overground train | Baseline | 15 | (21) | 15 | (19) | 8 | (12) | 9 | (14) | 16 | (23) | 20 | (22) | 17 | (25) | 12 | (12) |
|  | Follow-up | 14 | (18) | 12 | (16) | 9 | (13) | 9 | (14) | 15 | (18) | 14 | (17) | 16 | (21) | 17 | (19) |
|  |  |  |  |  |  |  |  |  |  |  |  |  |  |  |  |  |  |
| Underground train | Baseline | 14 | (17) | 15 | (18) | 12 | (20) | 8 | (15) | 15 | (17) | 21 | (18) | 16 | (16) | 19 | (15) |
|  | Follow-up | 12 | (15) | 15 | (17) | 8 | (12) | 10 | (14) | 14 | (17) | 18 | (18) | 13 | (14) | 24 | (17) |
|  |  |  |  |  |  |  |  |  |  |  |  |  |  |  |  |  |  |
| Stationary | Baseline | 440 | (193) | 449 | (188) | 507 | (192) | 496 | (184) | 421 | (190) | 418 | (179) | 407 | (184) | 380 | (207) |
|  | Follow-up | 461 | (216) | 215 | (156) | 531 | (201) | 183 | (148) | 434 | (225) | 233 | (163) | 441 | (198) | 279 | (128) |
|  |  |  |  |  |  |  |  |  |  |  |  |  |  |  |  |  |  |
| Total GPS minutes | Baseline | 552 | (207) | 558 | (195) | 602 | (201) | 596 | (190) | 537 | (206) | 537 | (188) | 529 | (210) | 482 | (225) |
|  | Follow-up | 570 | (229) | 316 | (183) | 629 | (208) | 280 | (177) | 546 | (242) | 336 | (188) | 557 | (218) | 395 | (136) |
|  |  |  |  |  |  |  |  |  |  |  |  |  |  |  |  |  |  |

**Footnotes**

1. Underground minutes are assumed from portions of missing GPS signal where the GPS signal is lost within 200m of an underground station and regained within 200m of a different underground station.

2. Stationary time is a mix of time spent stationary indoors and outdoors.
