# Supplementary material for: The effect of moving to East Village, the former London 2012 Olympic and Paralympic Games Athletes' Village, on mode of travel (ENABLE London study, a natural experiment)
Source: Int J Behav Nutr Phys Act. 2020 Feb 10;17:15. doi: 10.1186/s12966-020-0916-0 (PMC7011441; doi:10.1186/s12966-020-0916-0)
Supplement: Supplementary file 2 — Additional file 2: Table S2. Change in daily minutes of activity measured by GPS in East Village group relative to change in Control group, for those who were working or studying at baseline. [file 12966_2020_916_MOESM2_ESM.docx]

**S2 Table. Change in daily minutes of activity measured by GPS in East Village group relative to change in Control group, for those who were work or study at baseline**

|  | **All housing groups** | | | **Social** | | | **Intermediate** | | | **Market rent** | | |
| --- | --- | --- | --- | --- | --- | --- | --- | --- | --- | --- | --- | --- |
|  | **N=578** | | | **N=201** | | | **N=283** | | | **N=94** | | |
| **GPS motion category** | **Difference** | **(95% CI)** | **p-value** | **Difference** | **(95% CI)** | **p-value** | **Difference** | **(95% CI)** | **p-value** | **Difference** | **(95% CI)** | **p-value** |
| Walking |  |  |  |  |  |  |  |  |  |  |  |  |
| All participants | -1.4 | (-5.3, 2.5) | 0.48 | -1.8 | (-8.4, 4.8) | 0.59 | -1.9 | (-7.5, 3.8) | 0.52 | -4.2 | (-15.4, 7.0) | 0.46 |
| Work or study at baseline | -1.7 | (-5.8, 2.5) | 0.43 | 0.1 | (-7.7, 7.8) | 0.98 | -3.0 | (-8.7, 2.8) | 0.31 | -3.9 | (-15.7, 7.9) | 0.52 |
|  |  |  |  |  |  |  |  |  |  |  |  |  |
| Cycling |  |  |  |  |  |  |  |  |  |  |  |  |
| All participants | 1.1 | (-0.5, 2.7) | 0.17 | 0.3 | (-0.8, 1.4) | 0.58 | 1.0 | (-1.6, 3.7) | 0.44 | 1.9 | (-3.6, 7.4) | 0.50 |
| Work or study at baseline | 1.3 | (-0.6, 3.1) | 0.18 | 0.7 | (-0.3, 1.7) | 0.19 | 0.9 | (-1.9, 3.7) | 0.52 | 2.1 | (-3.7, 8.0) | 0.47 |
|  |  |  |  |  |  |  |  |  |  |  |  |  |
| Walking + cycling |  |  |  |  |  |  |  |  |  |  |  |  |
| All participants | -0.4 | (-4.7, 3.8) | 0.85 | -1.5 | (-8.4, 5.4) | 0.68 | -0.9 | (-7.1, 5.4) | 0.78 | -2.4 | (-16.0, 11.3) | 0.74 |
| Work or study at baseline | -0.5 | (-5.1, 4.1) | 0.84 | 0.9 | (-7.2, 9.0) | 0.83 | -2.0 | (-8.3, 4.4) | 0.55 | -1.0 | (-15.1, 13.2) | 0.89 |
|  |  |  |  |  |  |  |  |  |  |  |  |  |
| Motorised vehicle |  |  |  |  |  |  |  |  |  |  |  |  |
| All participants | -8.3 | (-14.0, -2.5) | 0.01 | -6.2 | (-18.9, 6.6) | 0.34 | -9.6 | (-16.9, -2.2) | 0.01 | -6.9 | (-19.9, 6.1) | 0.30 |
| Work or study at baseline | -5.7 | (-12.1, 0.8) | 0.09 | 0.9 | (-17.0, 18.9) | 0.92 | -8.2 | (-15.4, -1.1) | 0.02 | -7.6 | (-21.1, 5.8) | 0.27 |
|  |  |  |  |  |  |  |  |  |  |  |  |  |
| Overground train |  |  |  |  |  |  |  |  |  |  |  |  |
| All participants | -0.8 | (-3.7, 2.2) | 0.62 | -1.7 | (-5.8, 2.4) | 0.41 | -1.5 | (-6.0, 3.0) | 0.51 | 0.2 | (-10.1, 10.6) | 0.97 |
| Work or study at baseline | -1.5 | (-4.8, 1.8) | 0.37 | -2.8 | (-7.9, 2.3) | 0.29 | -2.7 | (-7.2, 1.8) | 0.24 | -0.5 | (-10.8, 9.8) | 0.93 |
|  |  |  |  |  |  |  |  |  |  |  |  |  |
| Underground train |  |  |  |  |  |  |  |  |  |  |  |  |
| All participants | 3.9 | (1.2, 6.5) | 0.005 | 4.0 | (-0.1, 8.1) | 0.06 | 1.6 | (-2.7, 6.0) | 0.46 | 11.5 | (4.4, 18.6) | 0.001 |
| Work or study at baseline | 3.0 | (0.0, 6.0) | 0.05 | 1.3 | (-3.6, 6.1) | 0.61 | 1.4 | (-3.1, 5.8) | 0.55 | 12.3 | (5.0, 19.7) | 0.001 |
|  |  |  |  |  |  |  |  |  |  |  |  |  |
| Stationary |  |  |  |  |  |  |  |  |  |  |  |  |
| All participants | -247 | (-279, -214) | <0.001 | -354 | (-405, -304) | <0.001 | -205 | (-252, -157) | <0.001 | -127 | (-217, -38) | 0.01 |
| Work or study at baseline | -217 | (-251, -182) | <0.001 | -298 | (-357, -239) | <0.001 | -200 | (-249, -152) | <0.001 | -127 | (-219, -34) | 0.01 |
|  |  |  |  |  |  |  |  |  |  |  |  |  |
| Total GPS minutes |  |  |  |  |  |  |  |  |  |  |  |  |
| All participants | -253 | (-288, -217) | <0.001 | -357 | (-412, -302) | <0.001 | -217 | (-269, -164) | <0.001 | -126 | (-223, -29) | 0.01 |
| Work or study at baseline | -222 | (-260, -184) | <0.001 | -294 | (-361, -228) | <0.001 | -214 | (-267, -161) | <0.001 | -125 | (-225, -24) | 0.01 |
|  |  |  |  |  |  |  |  |  |  |  |  |  |

**Footnotes**

1. All models are adjusted for sex, age group, ethnic group as fixed effects and household as a random effect in a multi-level model

2. The model for all housing group additionally adjusts for housing tenure group as a fixed effect.

3. Underground minutes are assumed from portions of missing GPS signal where the GPS signal is lost within 200m of an underground station and regained within 200m of a different underground station.

4. Across all housing groups, 485 (84%) participants were working or studying at baseline. The numbers for each housing group were 131 (65%), 266 (94%) and 88 (94%) for Social, Intermediate and Market-rent groups respectively.
