# Supplementary material for: The effect of moving to East Village, the former London 2012 Olympic and Paralympic Games Athletes' Village, on mode of travel (ENABLE London study, a natural experiment)
Source: Int J Behav Nutr Phys Act. 2020 Feb 10;17:15. doi: 10.1186/s12966-020-0916-0 (PMC7011441; doi:10.1186/s12966-020-0916-0)
Supplement: Supplementary file 3 — Additional file 3: Table S3. Change in daily minutes of activity measured by GPS in East Village group relative to change in Control group, for weekdays and weekend days. [file 12966_2020_916_MOESM3_ESM.docx]

**S3 Table. Change in daily minutes of activity measured by GPS in East Village group relative to change in Control group, for weekdays and weekend days.**

|  |  | **All housing groups** | | | **Social** | | | **Intermediate** | | | **Market rent** | | |
| --- | --- | --- | --- | --- | --- | --- | --- | --- | --- | --- | --- | --- | --- |
|  |  | **N=578** | | | **N=201** | | | **N=283** | | | **N=94** | | |
| **GPS motion category** | | **Difference** | **(95% CI)** | **p-value** | **Difference** | **(95% CI)** | **p-value** | **Difference** | **(95% CI)** | **p-value** | **Difference** | **(95% CI)** | **p-value** |
| Walking | All days | -1.4 | (-5.3, 2.5) | 0.48 | -1.8 | (-8.4, 4.8) | 0.59 | -1.9 | (-7.5, 3.8) | 0.52 | -4.2 | (-15.4, 7.0) | 0.46 |
|  | Weekdays | -2.7 | (-6.8, 1.4) | 0.20 | -1.5 | (-9.2, 6.1) | 0.69 | -4.4 | (-10.0, 1.2) | 0.13 | -4.1 | (-15.8, 7.7) | 0.50 |
|  | Weekend days | 2.2 | (-6.2, 10.6) | 0.61 | -2.2 | (-11.8, 7.5) | 0.66 | 2.5 | (-11.4, 16.4) | 0.72 | -7.3 | (-32.3, 17.7) | 0.57 |
|  |  |  |  |  |  |  |  |  |  |  |  |  |  |
| Cycling | All days | 1.1 | (-0.5, 2.7) | 0.17 | 0.3 | (-0.8, 1.4) | 0.58 | 1.0 | (-1.6, 3.7) | 0.44 | 1.9 | (-3.6, 7.4) | 0.50 |
|  | Weekdays | 1.1 | (-0.6, 2.8) | 0.20 | 0.2 | (-1.2, 1.5) | 0.82 | 2.2 | (-0.7, 5.1) | 0.14 | -0.2 | (-6.2, 5.7) | 0.94 |
|  | Weekend days | 0.4 | (-3.5, 4.3) | 0.84 | 0.3 | (-0.8, 1.5) | 0.55 | -2.1 | (-8.3, 4.1) | 0.51 | 6.0 | (-9.2, 21.1) | 0.44 |
|  |  |  |  |  |  |  |  |  |  |  |  |  |  |
| Walking + cycling | All days | -0.4 | (-4.7, 3.8) | 0.85 | -1.5 | (-8.4, 5.4) | 0.68 | -0.9 | (-7.1, 5.4) | 0.78 | -2.4 | (-16.0, 11.3) | 0.74 |
|  | Weekdays | -2.1 | (-6.5, 2.3) | 0.34 | -1.6 | (-9.4, 6.2) | 0.68 | -2.7 | (-8.8, 3.4) | 0.38 | -5.7 | (-19.8, 8.3) | 0.42 |
|  | Weekend days | 3.0 | (-6.1, 12.1) | 0.52 | -1.5 | (-11.5, 8.4) | 0.76 | 0.04 | (-14.6, 14.7) | 1.00 | -0.7 | (-30.2, 28.8) | 0.96 |
|  |  |  |  |  |  |  |  |  |  |  |  |  |  |
| Motorised vehicle | All days | -8.3 | (-14.0, -2.5) | 0.01 | -6.2 | (-18.9, 6.6) | 0.34 | -9.6 | (-16.9, -2.2) | 0.01 | -6.9 | (-19.9, 6.1) | 0.30 |
|  | Weekdays | -7.8 | (-14.4, -1.2) | 0.02 | -3.1 | (-18.3, 12.2) | 0.69 | -10.6 | (-18.7, -2.5) | 0.01 | -4.6 | (-17.2, 8.0) | 0.47 |
|  | Weekend days | -11.9 | (-22.0, -1.9) | 0.02 | -15.8 | (-33.7, 2.1) | 0.08 | -9.6 | (-24.4, 5.2) | 0.21 | -27.2 | (-55.5, 1.1) | 0.06 |
|  |  |  |  |  |  |  |  |  |  |  |  |  |  |
| Overground train | All days | -0.8 | (-3.7, 2.2) | 0.62 | -1.7 | (-5.8, 2.4) | 0.41 | -1.5 | (-6.0, 3.0) | 0.51 | 0.2 | (-10.1, 10.6) | 0.97 |
|  | Weekdays | -0.6 | (-4.0, 2.9) | 0.74 | -2.7 | (-7.4, 2.0) | 0.27 | -1.4 | (-6.9, 4.0) | 0.61 | 1.5 | (-8.9, 11.9) | 0.78 |
|  | Weekend days | -1.9 | (-6.4, 2.6) | 0.40 | 1.6 | (-2.4, 5.6) | 0.43 | -3.3 | (-9.9, 3.3) | 0.33 | -11.3 | (-29.2, 6.7) | 0.22 |
|  |  |  |  |  |  |  |  |  |  |  |  |  |  |
| Underground train | All days | 3.9 | (1.2, 6.5) | 0.005 | 4.0 | (-0.1, 8.1) | 0.06 | 1.6 | (-2.7, 6.0) | 0.46 | 11.5 | (4.4, 18.6) | 0.001 |
|  | Weekdays | 3.1 | (0.03, 6.2) | 0.048 | 2.2 | (-2.0, 6.5) | 0.30 | 0.8 | (-4.4, 6.1) | 0.75 | 13.0 | (4.9, 21.1) | 0.002 |
|  | Weekend days | 4.6 | (0.2, 9.0) | 0.04 | 6.2 | (-1.8, 14.2) | 0.13 | 4.2 | (-2.4, 10.7) | 0.21 | 2.1 | (-8.3, 12.6) | 0.69 |
|  |  |  |  |  |  |  |  |  |  |  |  |  |  |
| Stationary | All days | -247 | (-279, -214) | <0.001 | -354 | (-405, -304) | <0.001 | -205 | (-252, -157) | <0.001 | -127 | (-217, -38) | 0.01 |
|  | Weekdays | -238 | (-273, -203) | <0.001 | -344 | (-397, -290) | <0.001 | -196 | (-248, -144) | <0.001 | -125 | (-225, -26) | 0.01 |
|  | Weekend days | -313 | (-357, -268) | <0.001 | -409 | (-483, -336) | <0.001 | -262 | (-330, -195) | <0.001 | -207 | (-315, -98) | <0.001 |
|  |  |  |  |  |  |  |  |  |  |  |  |  |  |
| All GPS minutes | All days | -253 | (-288, -217) | <0.001 | -357 | (-412, -302) | <0.001 | -217 | (-269, -164) | <0.001 | -126 | (-223, -29) | 0.01 |
|  | Weekdays | -246 | (-284, -208) | <0.001 | -347 | (-407, -287) | <0.001 | -211 | (-268, -154) | <0.001 | -122 | (-228, -15) | 0.03 |
|  | Weekend days | -318 | (-368, -268) | <0.001 | -418 | (-500, -337) | <0.001 | -275 | (-353, -197) | <0.001 | -236 | (-354, -118) | <0.001 |
|  |  |  |  |  |  |  |  |  |  |  |  |  |  |

**Footnotes**

1. Data for weekdays only were available for 569 participants overall and for 198, 277 and 94 participants in the social, intermediate and market-rent groups respectively. Data for weekend days only were available for 383 participants and for 125, 194 and 64 participants in the social, intermediate and market-rent groups respectively.

2. All models are adjusted for sex, age group, ethnic group as fixed effects and household as a random effect in a multi-level model

3. The model for all housing group additionally adjusts for housing tenure group as a fixed effect.

4. Underground minutes are assumed from portions of missing GPS signal where the GPS signal is lost within 200m of an underground station and regained within 200m of a different underground station.
